# The development of an artificial neural network – genetic algorithm model (ANN-GA) for the adsorption and photocatalysis of methylene blue on a novel sulfur–nitrogen co-doped Fe_2_O_3_ nanostructure surface

**DOI:** 10.1039/c9ra10349j

**Published:** 2020-02-05

**Authors:** Roya Mohammadzadeh Kakhki, Mojtaba Mohammadpoor, Reza Faridi, Mehdi Bahadori

**Affiliations:** Department of Chemistry, Faculty of Sciences, University of Gonabad Gonabad Iran Romohammadzadeh@gonabad.ac.ir; Electrical and Computer Eng. Department, University of Gonabad Gonabad Iran

## Abstract

In this study, a new sulfur–nitrogen co-doped Fe_2_O_3_ nanostructure was synthesized *via* a simple and efficient method and characterized *via* UV-Vis spectrophotometry, X-ray diffraction, field emission scanning electron microscopy, energy-dispersive X-ray spectroscopy, and Brunauer–Emmett–Teller surface area analysis. The as-synthesized nanoparticles showed high efficiency for the removal of methylene blue. The experimental conditions including the dose of the nanoparticle, the concentration of the dye, pH and the light dose were studied and optimized. The removal percentage was approximately 95% in a short time (5 min). A three-layer artificial neural network (ANN) model was proposed for predicting the efficiency of the dye removal. The network was trained using the obtained experimental data at optimum values. Some training functions were tested and their ability to predict different numbers of neurons was evaluated. The coefficient of determination (*R*-squared) and the mean squared error (MSE) were measured for comparison. In order to improve the accuracy of the prediction and to remove its dependency on the number of neurons, the ANN parameters were optimized using the genetic algorithm (GA). The final model results showed an acceptable agreement with experimental data. Furthermore, the relative importance of the dose of the nanoparticle, the concentration of the dye, and pH on the efficiency were obtained as 39%, 46%, and 15%, respectively. Moreover, interestingly, the obtained results showed that this newly synthesized nanoparticle has some photocatalytic properties with a band gap of 1.65 eV and therefore, it can be proposed as a low-cost visible light-driven photocatalyst for engineering applications.

## Abstract 2

In this research an S-N doped Fe_2_O_3_ nanostructure is synthesized and its adsorption ability and photocatalytic activity were evaluated. The optimum experimental conditions were obtained and an ANN-GA model was proposed for predicting experimental values.

## Graphical abstract

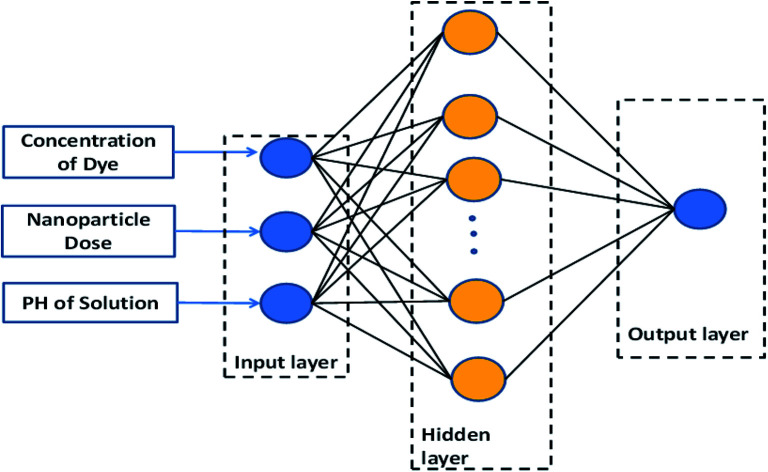

## 1. Introduction

Fe_2_O_3_ is a unique material with special characteristics such as high stability, oxidizing power, low cost, environmental friendly, and availability, and it also has specific visible light features. Therefore, developing a simple and low-cost method for synthesizing this nanoparticle is very interesting. The synthesis method affects the surface area and sorption capability. Some of the previous studies have been focused on the application of Fe_2_O_3_ and doped Fe_2_O_3_ in the removal of pollutants, but they have some disadvantages, such as the high-cost of the synthetic methods.^1^

In addition to sorption features, nanoparticles can have some photocatalytic properties related to the synthesis method and the surface structure. The degradation of dye pollutants with a photocatalyst is the best way to remove contaminants permanently. Among the materials with this property, visible light-driven photocatalysts have received increasing attention due to their low-cost and clean energy. The photocatalytic property is due to the absorption of light and electron–hole separation. Therefore, some charge carriers are produced. For materials with a low bandgap energy, light of low energy, such as visible light, can excite the photocatalyst. In the case of iron oxide, the bandgap is about 2.2 eV; however, the probability of carrier recombination is an issue. The type of nanocrystal used and doping with heteroatoms affects the photocatalytic performance of nanomaterials.^2–4^ Doping of other atoms such as nitrogen and sulfur results in changes in the chemical, physical, and redox properties of the materials.^5–7^ Doping these non-metal atoms causes mixing of the p orbitals of oxygen and the non-metal atoms, leading to an adjusted bandgap. It is well known that the presence of sulfur atoms in photocatalysts, by creating a trap state (separate band) in between the valence band and conduction band, leads to the slower recombination of the carrier charge and therefore causes the photocatalysts to preform higher.^8^

Methylene blue (MB) is a dye with a heterocyclic structure that has various applications but its toxicity causes some problems for plants and animals. Therefore, investigations on the removal and degradation of MB are very interesting.^9–13^ A suitable photocatalyst can help the degradation of the pollutant to yield a clean environment. Among the reported nanophotocatalysts, noble-metal-free catalysts are very interesting due to their availability, low-cost and unique benefits.^14–18^ Fe_2_O_3_ is an interesting material that can be used as an alternative material for this purpose.^19,20^ To date, Fe_2_O_3_, as an n-type nanomaterial, has been synthesized *via* various methods^21–24^ but most of them are not affordable. Among the various methods, the co-precipitation method is a simple and low-cost method that has been widely used in various studies.

In order to consider the interactions of variables that influence this process, modeling and optimization of variables must be performed by minimum experiments. One of the most powerful tools for modeling linear and non-linear systems is artificial neural networks (ANN).^25^ A genetic algorithm (GA) is used for optimizing the ANN parameters. ANN and its GA-optimized version have been widely used in various research areas. Ghaedi *et al.* have proposed a tree layer ANN-GA for the accurate prediction of the dye removal percentage of graphite oxide from aqueous solutions. They were in good agreement with experimental data. Hassani *et al.* also proposed an ANN-GA model to determine the optimum conditions of initial pH, ozone flow rate, initial ciprofloxacin concentration, catalyst dosage, and reaction time for degradation efficiency. Their results showed an acceptable agreement between the GA predicted and experimental efficiencies.^26^

In this research, a sulphur and nitrogen co-doped Fe_2_O_3_ nanostructure was synthesized *via* a simple co-precipitation method. The adsorption ability and photocatalytic activity of the as-synthesized nanomaterial were evaluated. The optimum experimental conditions were obtained and an ANN-GA model was proposed for predicting experimental values.

## 2. Experimental section

### 2.1 Materials

All the chemical materials were purchased from Merck and Sigma companies and were used without further purification.

### 2.2 Synthesis methods

4 mmol of iron nitrate and 4 mmol of thiourea were dissolved in 20 mL of distilled water and then 3 mL of ethanol was added and the reaction was stirred. Then, about 0.5 mL of ammonium hydroxide was added to the above solution. The solution was stirred vigorously and after 60 min the as-prepared precipitate was separated and washed with distilled water and ethanol. Finally, it was dried in an oven at 80 °C and sintered at 300 °C for 3 h.

### 2.3 Removal experiments

For evaluating the removal performance of MB with this new nanostructure, about 15 mg of the nanoparticles were added to 40 mL of MB (5 ppm) and after the indicated times samples were taken out. After the separation of the nanoparticles, the samples were measured *via* UV-Vis spectrophotometry. In each case, the removal percent was calculated using the following equation:

1 Removal% = (*A*_0_ − *A*/*A*_0_) × 100

where *A*_0_ and *A* are initial absorbance and current absorbance at each time, respectively.

Moreover, to investigate the optimum conditions, some experiments were performed and the effects of time, dose of nanoparticle, concentration, pH, and visible light irradiation was investigated.

To study the effects of light irradiation, a homemade photo-reactor was constructed, and its walls were mirrored. A low consume florescence of 60 W was used as the source.

### 2.4 ANN-GA model

**Fig. 1:**
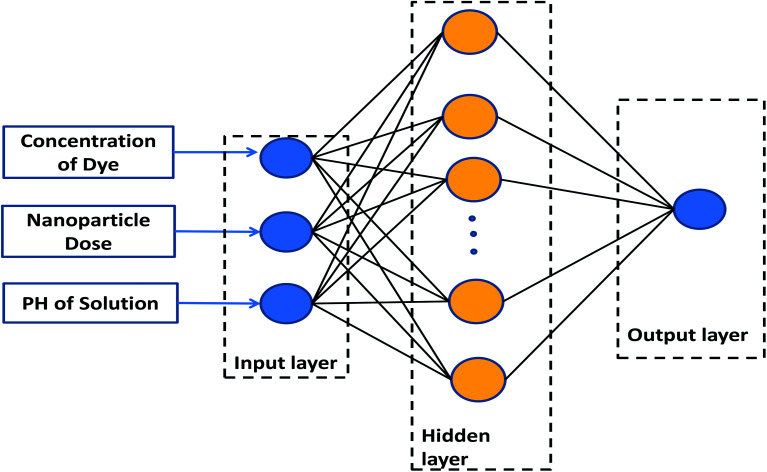
Proposed ANN model.

Artificial neural networks (ANN) are data processing techniques designed based on the biological neuron processing.^27,28^ Due to the ability of these systems to discern the relationship between inputs and outputs, they are useful tools in predicting the influence of operating parameters on chemical processes.^29^ An ANN model is comprised of input, hidden, and output layers, which should be managed in a proper manner.^30^Fig. 1 presents the proposed model, consisting of a three-layer ANN with a tangent sigmoid transfer function (tansig) at the hidden layer and a linear transfer function (purelin) at the output layer. Some different backpropagation algorithms exist, such as the Levenberg–Marquardt, Bayesian regularization, and resilient backpropagation algorithms. Their effects were evaluated in this research. The number of neurons in the hidden layer was optimized to be between 1–20 neurons. The characteristics of the input and output variables used in this research are shown in Table 1.

**Table 1:** Characteristics of input and output variables

Variable	Range
**Input layer**	
Concentration of dye	2–10 ppm
Nanoparticle dose	0.005–0.015 g
PH of solution	3–12

**Output layer**	
Normalized efficiency	0–1

The quality and reliability of an ANN are strongly dependent on the configuration of the network. For overcoming this, the parameters of the ANN are optimized using the genetic algorithm (GA).

GA is a metaheuristic method for solving optimization problems. It is a population-based algorithm defined based on Darwin's evolutionary technique that presents the idea of “survival of the fittest” and “natural selection”. It begins with a set of solutions, known as chromosomes, and encourages them to survive and reproduce the answers. By repeating this procedure an optimum solution may be reached.^31^

The data obtained from the experimental values were normalized between 0 and 1 to avoid numerical overflows.^25^ The following formulas were used to perform the normalization method:

2 *y*_*i*_ = (*x*_*i*_ − *x*_min_)/(*x*_max_ − *x*_min_)

where *y*_*i*_ is the normalized value of *x*_*i*_, and *x*_max_ and *x*_min_ are the maximum and minimum values of *x*_*i*_, respectively.

The results of different networks were evaluated based on the mean squared error (MSE) and the coefficient of determination (*R*-squared), which can be defined as follows:^25^3
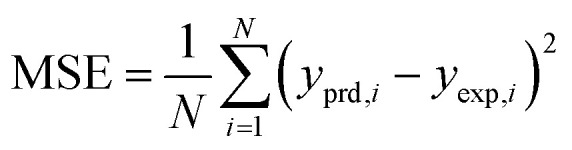
4
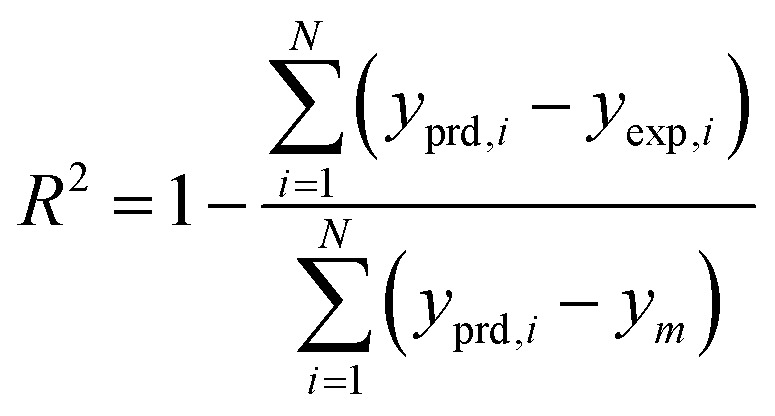
where *N*, *y*_prd, *i*_, *y*_exp, *i*_ and *y*_m_ are the number of data, the *i*th models predicted value, the *i*th experimental value, and the average of the experimental values, respectively.

## 3. Results and discussion

### 3.1 XRD

**Fig. 2:**
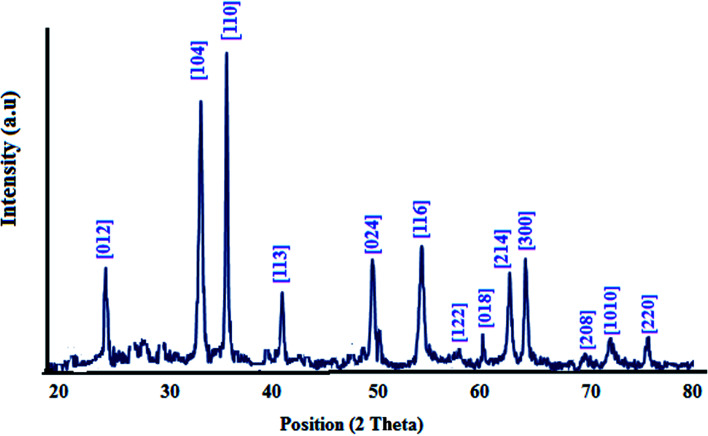
XRD pattern of the nanostructure.

XRD analysis of the as-synthesized nanomaterial is performed and is illustrated in Fig. 2. The patterns were indexed by the characteristic peaks (01-084-0308). Also the peak [110] is stronger than [104], showing doping of nitrogen and sulfur in this nanostructure, while for S doped or N doped nanostructures, [110] is along the [104] plane.^32^

### 3.2 FESEM and EDS analysis

**Fig. 3:**
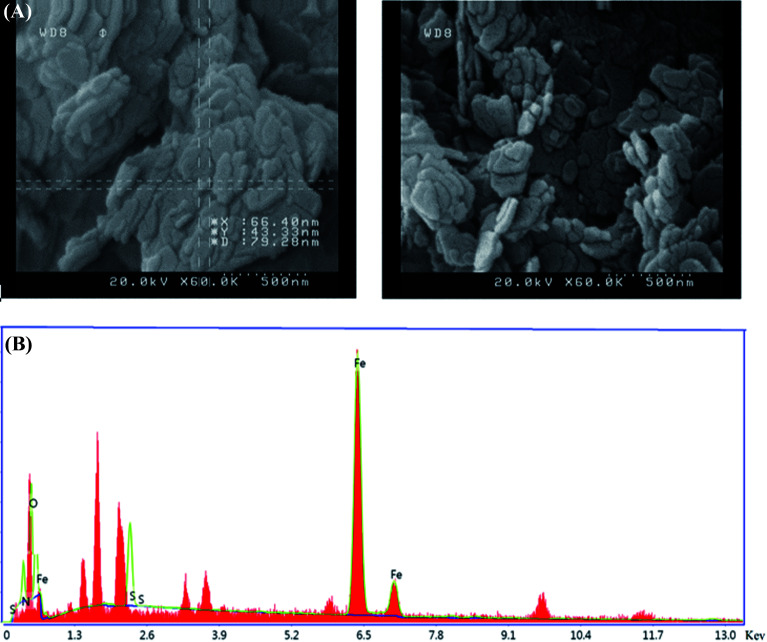
(A) FESEM image and (B) EDS analysis of the nanostructure.

As shown in the FESEM images in Fig. 3, the as-synthesized nanoparticles are on the nanoscale and their morphology is a nanoplate. The EDS analysis is also shown in this figure. It is clear that this nanoparticle contains Fe, O, N, and S atoms. Moreover, the percentage of nitrogen and sulphur are reported in Table 2.

**Table 2:** The EDS data for S,N co-doped Fe_2_O_3_

Element	Weight%	Atomic%
Fe	67.1	45.9
O	11.4	27.1
N	14.6	17.7
S	6.9	9.3

### 3.3 The BET surface areas and pore size distributions

**Fig. 4:**
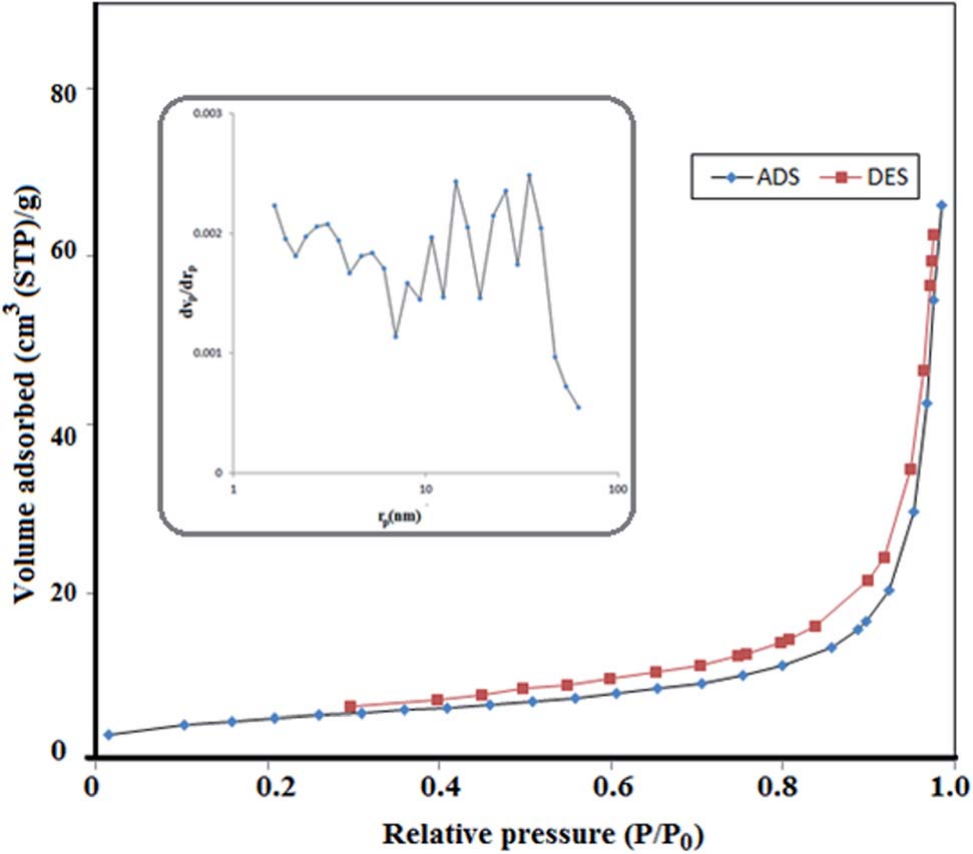
Nitrogen adsorption–desorption isotherms and the corresponding pore size distribution curves (inset) of S,N doped Fe_2_O_3_.

In order to study the BET surface areas and pore size distributions of the S,N co-doped Fe_2_O_3_ nanoparticles, N_2_ adsorption–desorption was performed. Nitrogen adsorption–desorption isotherms and pore size distributions are shown in Fig. 4, with an inset. The specific area of the proposed nanoparticle is about 17.5 m^2^ g^−1^ and it obeys a type-III isotherm. Moreover, it contains a wide range of pore sizes from 0.1 to 46 nm with an average pore diameter of 23.341 nm and a total pore volume of 0.1021 cm^3^ g^−1^. Therefore, due to its high surface area, porosity and small pores, the active sites enhanced and lead to a high photocatalytic activity.^33^

### 3.4 Removal studies

To investigate the ability of the nanoparticles for removing pollutants, methylene blue was selected as a reprehensive sample and some experimental conditions were studied.

#### 3.4.1 Effect of nanoparticle dose and time of removal

**Fig. 5:**
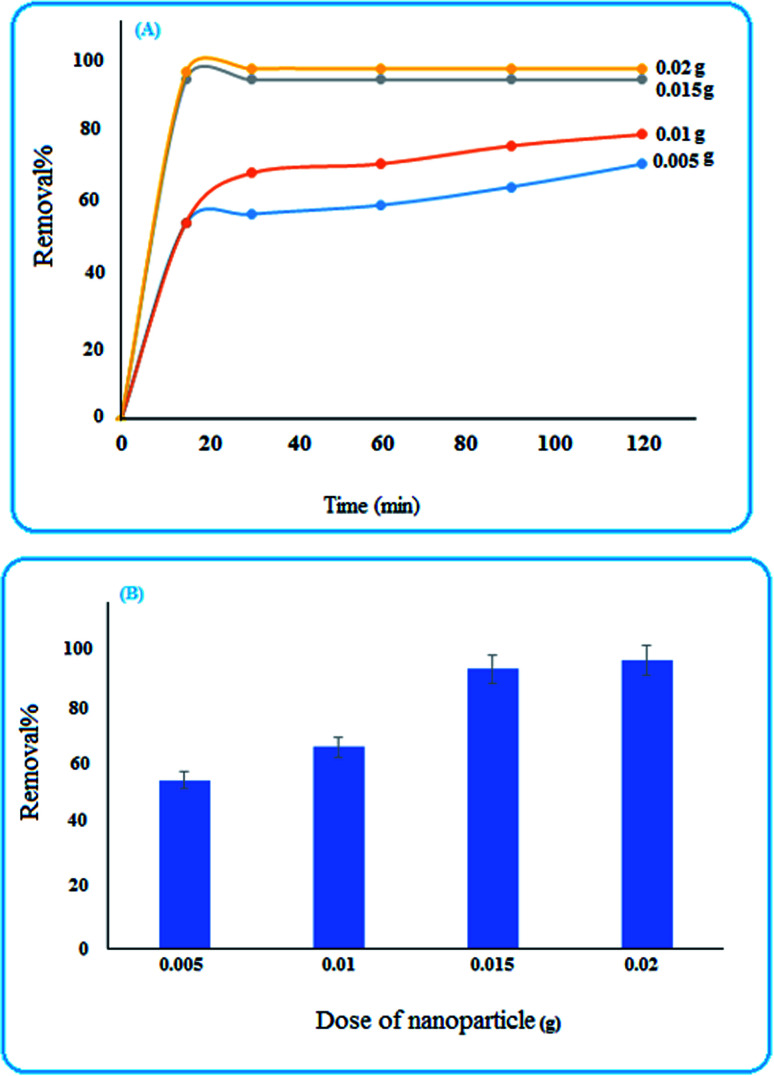
(A) Influence of time and the dose of the nanoparticle on the removal percentage, (B) removal efficiency of MB after 5 min.

To investigate the effects of nanoparticle concentration and time on the removal efficiency, some doses (0.005, 0.01, 0.015, and 0.02 g) of S,N doped Fe_2_O_3_ were examined after 120 min. As can be observed in Fig. 5(A), with the increase in the nanoparticle concentration, the removal percentage enhanced. Also, most removal was obtained in the initial 5 min; therefore, the efficiency was studied over 5 min. The results in Fig. 5(B) show that by increasing the amount of catalyst to 0.015 g the efficiency increased and then remained constant. Therefore, 0.015 g was selected for further investigations.^12,13^

#### 3.4.2 Effects of concentration

**Fig. 6:**
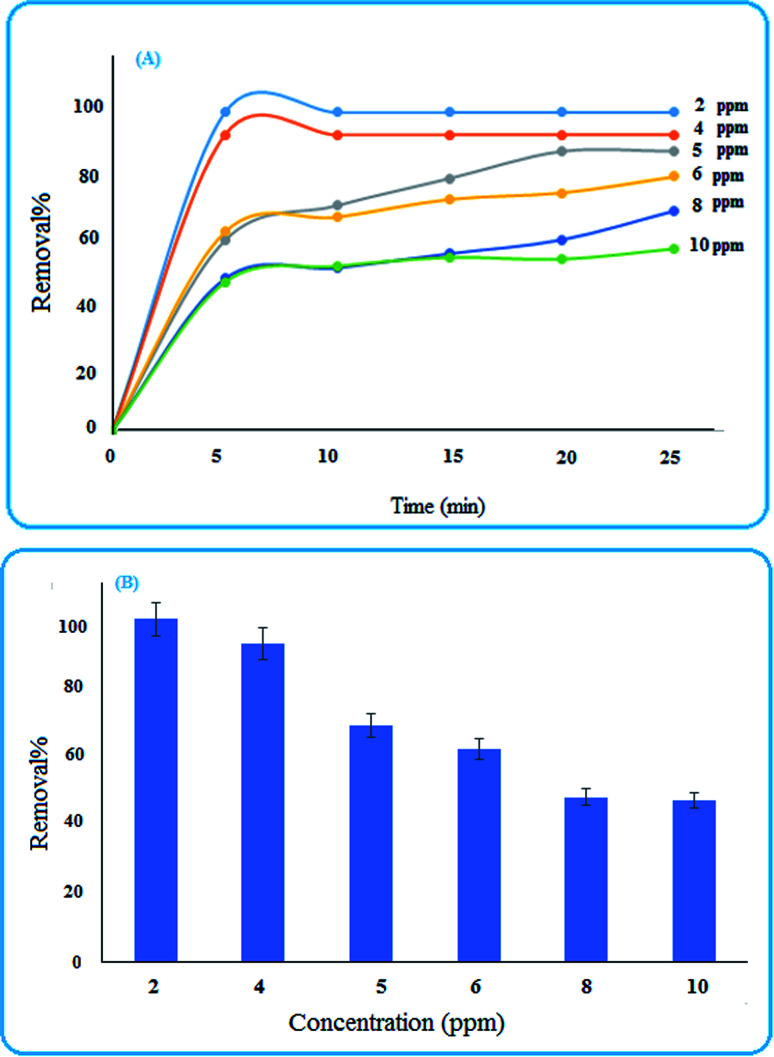
(A) Influence of time and dye concentration on the removal percentage (B) removal efficiency of MB after 5 min.

Various concentrations (2, 4, 5, 6, 8, and 10 ppm) of the dye solution were tested, and the results illustrated in Fig. 6 show that with the increase in the concentration, the removal efficiency decreased. This may be due to the occupation of surface sites with dye molecules. Also, the removal percentage at different concentrations of MB in the initial 5 min are shown in Fig. 6(B).^12,13^

#### 3.4.3 Effect of solution pH

**Fig. 7:**
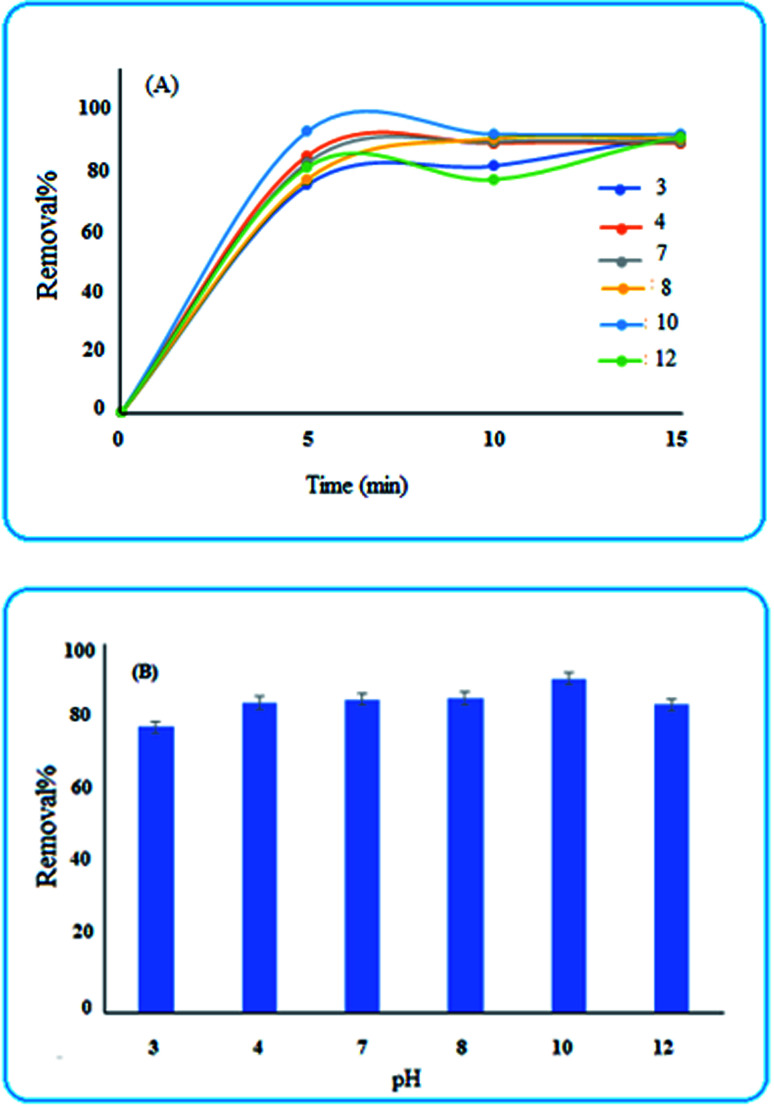
(A) Influence of time and pH on the removal percentage (B) removal efficiency of MB after 5 min.

The pH of the solution plays a critical role in the removal percentage. For evaluating this, some acidic and basic solutions were tested. As shown in Fig. 7, the efficiency over 5 min, is relatively low for pH values lower than 4. This is due to the fact that for pH values lower than the isoelectric point (*i.e.*, 4.24) the surface charge of Fe_2_O_3_ is positive^34^ and therefore the adsorption of MB will be decreased. Moreover, for pH values higher than 4.24, the surface charge will be negative, and hence, the adsorption of the positively charged dye is more efficient on the surface of the nanosorbent.^12,13^

### 3.5 ANN-GA predictions

**Fig. 8:**
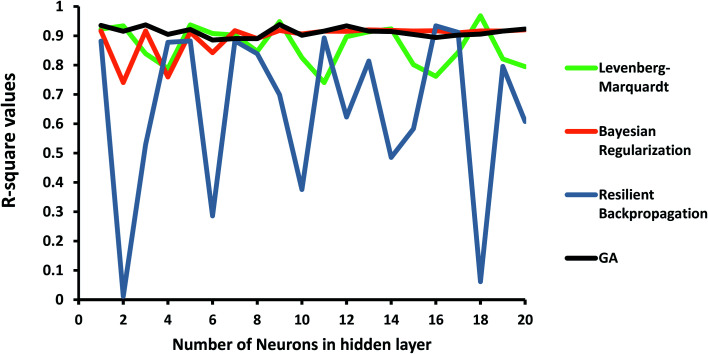
*R*-squared values at different numbers of neurons and propagation algorithms.

**Fig. 9:**
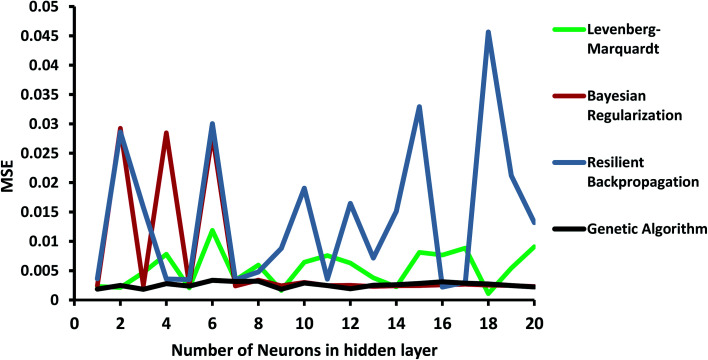
MSE at different numbers of neurons and propagation algorithms.

In order to investigate the ANN model, the structure shown in Fig. 1 was launched using the experimental data, including concentration, pH of the solution, and the dose of the nanoparticle as network inputs, and the removal efficiency of MB as the output. The different number of neurons in the hidden layer as well as some different backpropagation techniques, namely Levenberg–Marquardt, Bayesian regularization, and resilient backpropagation, were evaluated. *R*-squared values and MSEs at different numbers of neurons are shown in Fig. 8 and 9, respectively.

As shown in Fig. 8, the performance of the ANN network is highly dependent on the backpropagation technique and the number of neurons in the hidden layer. Some algorithms, such as the resilient backpropagation algorithm, are more sensitive and will not improve with an increasing number of neurons. Some of them including Bayesian regularization are more stable and show acceptable performance at a higher number of neurons.

In order to improve the performance of the model, the ANN parameters were optimized using the genetic algorithm. *R*-squared and MSE values are also shown in Fig. 8 and 9. The ANN-GA model is robust and achieves the best performance, generally around 0.91 and 0.003 for the *R*-squared value and MSE, respectively. These values imply acceptable predictions when compared to experimental values.

### 3.6 Measuring the relative importance of each input variable

**Fig. 10:**
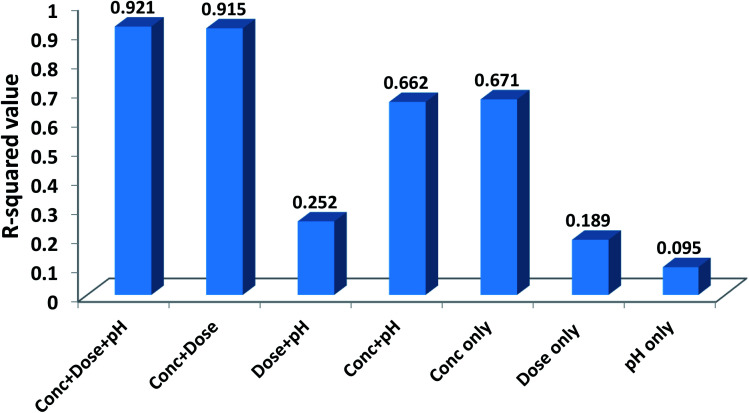
Effects of different input selections on efficiency.

In order to evaluate the contribution of each of the inputs (concentration, the dose of the nanoparticle, and pH of the solution) on the prediction results, the model was launched again with varying input selections. The *R*-squared and MSE values are shown in Fig. 10. As implied, omitting pH from the consideration has a minor effect on the results and reduces the *R*-squared value from just 0.921 to 0.915. In contrast, by selecting only concentration as the ANN input, the *R*-squared value will reach 0.671.

**Fig. 11:**
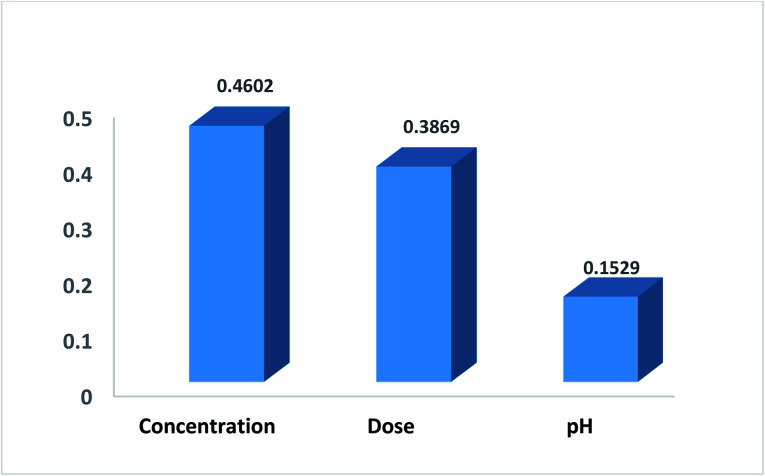
The relative importance of each input variable.

Garson^35^ proposed an algorithm for measuring the relative importance of an input variable on a modeling network. In the case of a single layer of hidden units, the equation is:5
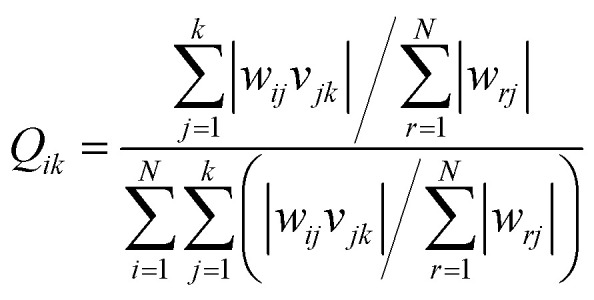
where *w*_*ij*_ is the weight between the *i*th input and the *j*th hidden unit and *v*_*jk*_ is the weight between the *j*th hidden unit and the *k*th output. This method has been used by many researchers.^36,37^Fig. 11 shows the relative contribution of each input on the output. In this case, the number of neurons in the hidden layer is considered as 5 and GA was applied to the ANN. The results show that concentration (46%) and pH (15.2%) have the highest and lowest contribution, respectively, to the prediction output.

### 3.7 Study of photocatalytic activity of the nanomaterial

**Fig. 12:**
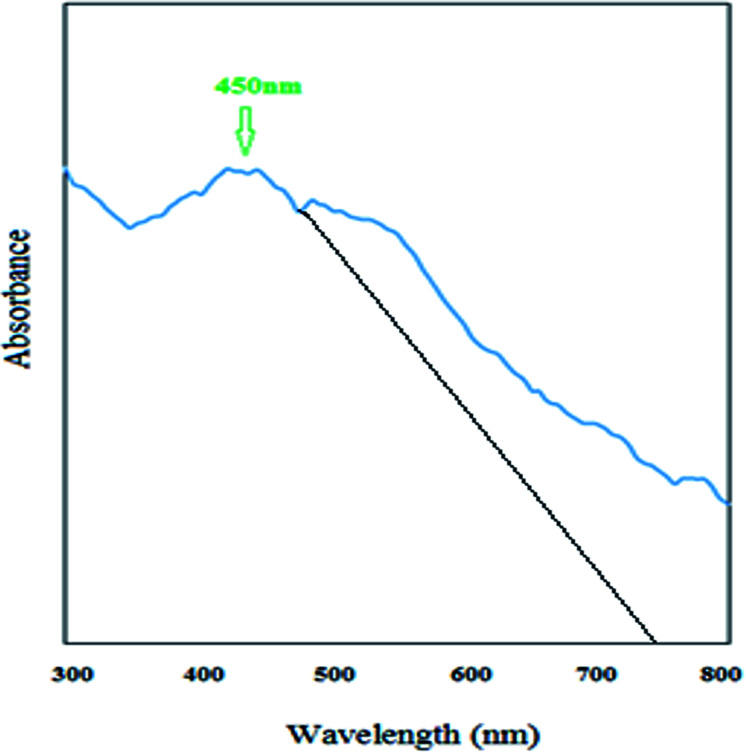
UV-Vis spectrum of S,N co-doped Fe_2_O_3_ nanostructures.

As can be observed from Fig. 12, the newly synthesized nanostructure has a broad peak in the visible region at a wavelength of 450 nm. Therefore, this new S–N doped iron oxide nanoparticle has some photonic properties and can be used in related photoelectronic works. As can be observed from Fig. 1, the cut off wavelength of the peak^38^ is at 750 nm and therefore, the energy of the band gap of this nanomaterial was calculated using Einstein's energy equation:

6 *E*_g_ = *hc*/*λ*

where *E*_g_ is the energy of band gap, *c* is the speed of light, *h* is the Planck's constant, and *λ* is the cutoff wavelength (Fig. 12). Based on this equation the band gap for this new nanostructure was found to be 1.65 eV; therefore, this nanomaterial is a visible light-driven photocatalyst.

**Fig. 13:**
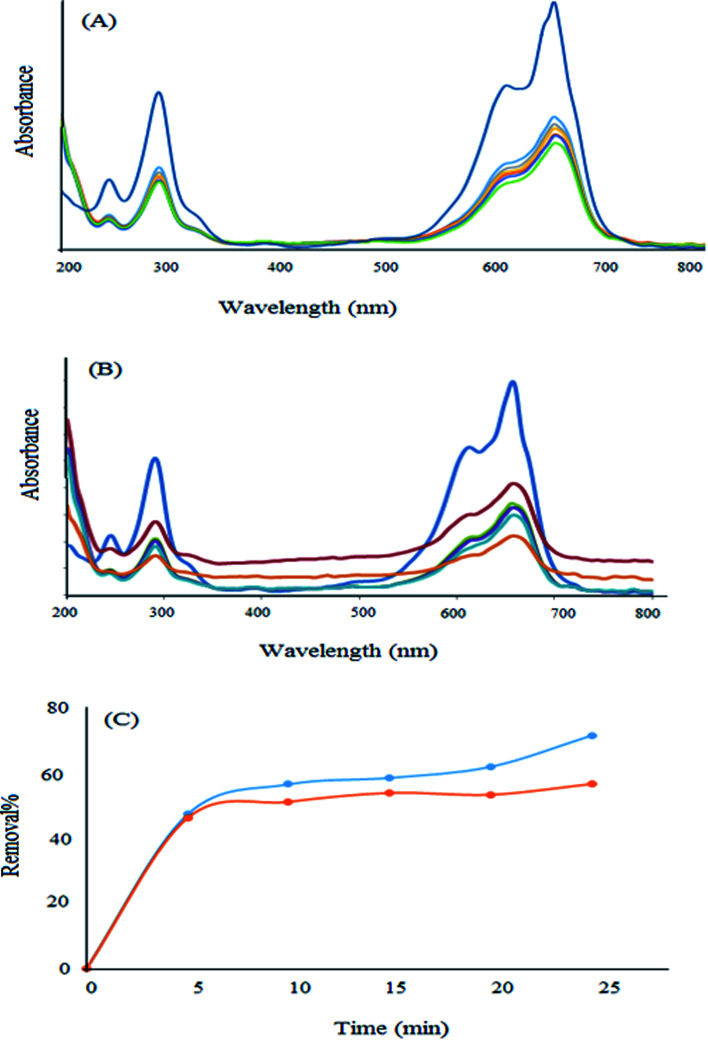
Removal of MB in (A) the absence and (B) the presence of light. (C) Comparison of the removal percentage in the presence and absence of light.

In order to investigate the photocatalytic properties of the newly synthesized nanomaterial, some experiments were performed in the presence of light. For this purpose, a high concentration (10 ppm) was selected and the removal percentage in the absence and presence of light was investigated. As can be observed from Fig. 13, the removal percentage enhanced to about 17% in the presence of visible light after 25 min. Therefore, this new nanomaterial has some photocatalytic properties in addition to its high surface area, which are advantageous for the efficient adsorption of pollutants. Moreover, they can degrade the dye molecule into less toxic substances.

Since the band gap of the as-synthesized nanoparticle is low (1.65 eV), this nanophotocatalyst can adsorb and degrade methylene blue under visible light and may do so under the light of the laboratory.

**Fig. 14:**
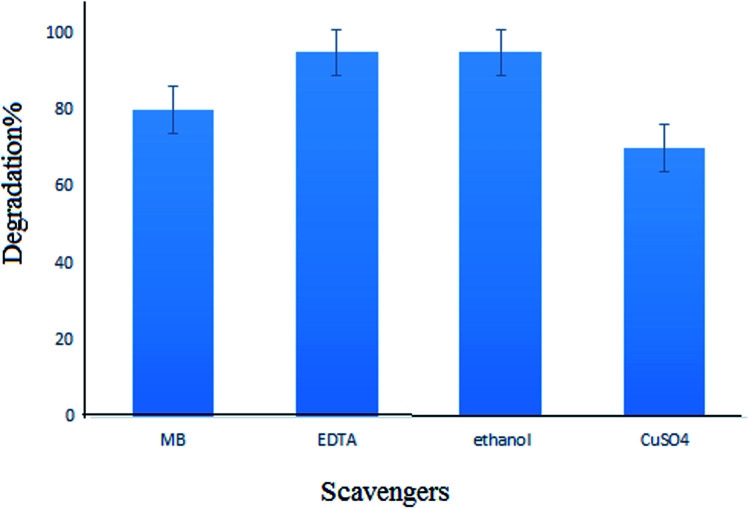
Effect of scavengers on the photodegradation of MB.

**Fig. 15:**
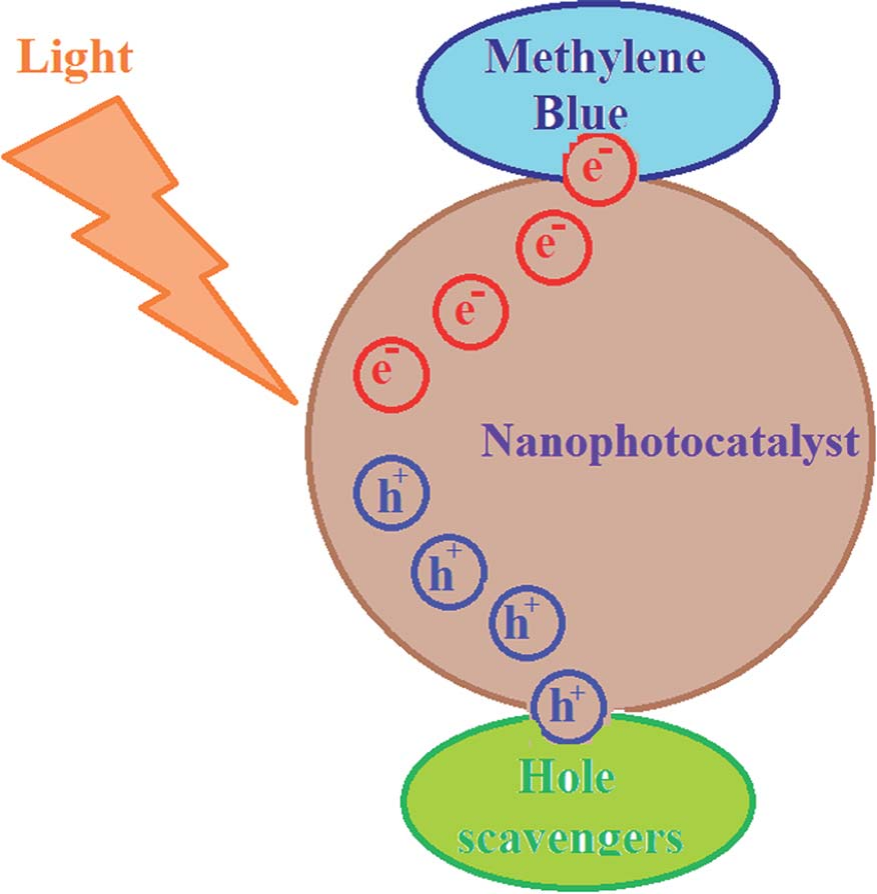
The mechanism for the photodegradation of methylene blue.

For investigating the photodegradation of MB with S,N-doped Fe_2_O_3_, some hole and electron scavengers were used. In this study, EDTA and propanol were used as hole scavengers and Cu^2+^ was used as an electron scavenger along with a photocatalyst. As shown in Fig. 14, the presence of hole scavengers leads to an increase in the photodegradation. This may be due to the separation of the pair (electron–hole) caused by the scavenging of the photogenerated holes.^39,40^ However, the addition of electron scavengers caused a decrease in the photodegradation efficiency. Thus, a photoreduction mechanism is proposed for this process (Fig. 15).

## 4. Conclusion

In this study, a new S,N co-doped nano-iron oxide material was synthesized *via* a simple co-precipitation method and was characterized. This new nanoparticle was applied successfully for removing methylene blue (as a representative of color pollutants), and the experimental conditions including the dose of the nanoparticle, concentration, pH and light effects were investigated. Also, the studies showed that the proposed nanomaterial has some photocatalytic effects that can be applied to removing organic dyes. A model is proposed for predicting experimental results based on artificial neural networks. The impact of different training algorithms and the number of neurons in the hidden layers were evaluated. The results showed high sensitivity to the adjusted parameters. To have an accurate and robust model, the ANN parameters were optimized using the genetic algorithm. The proposed ANN-GA model achieved acceptable performance, where the *R*-squared value was around 92%. Moreover, the relative importance of each input parameter on the prediction was evaluated and the results showed that concentration (46%) had the highest effect on the removal process. Moreover, the band gap of the newly synthesized photocatalyst was calculated to be about 1.65 eV. The effects of some hole and electron scavengers were investigated and a photoreduction mechanism was proposed for this process.

## Conflicts of interest

There are no conflicts to declare.

## Supplementary Material
